# Belowground plant competition: uncoupling root response strategies of peas

**DOI:** 10.1098/rspb.2024.0673

**Published:** 2024-07-31

**Authors:** Ruth Gottlieb, Michal Gruntman

**Affiliations:** ^1^ School of Plant Sciences and Food Security, Tel Aviv University, Tel Aviv, Israel; ^2^ Porter School of the Environment and Earth Sciences, Tel Aviv University, Tel Aviv, Israel

**Keywords:** belowground competition, nutrient heterogeneity, plant–plant interactions, *Pisum sativum*, root allocation, root placement

## Abstract

Belowground plant competition has been shown to induce varying responses, from increases to decreases in root biomass allocation or in directional root placement. Such inconsistencies could result from the fact that root allocation and directional growth were seldom studied together, even though they might represent different strategies. Moreover, variations in belowground responses might be due to different size hierarchies between plants, but this hypothesis has not been studied previously. In a greenhouse rhizobox experiment, we examined the way both root allocation and directional root placement of *Pisum sativum* are affected by the size and density of *Festuca glauca* neighbours, and by nutrient distribution. We found that root allocation of *P. sativum* increased with the density and size of *F. glauca*. By contrast, directional root placement was unaffected by neighbour size and increased either towards or away from neighbours when nutrients were patchily or uniformly distributed, respectively. These results demonstrate that directional root placement under competition is contingent on the distribution of soil resources. Interestingly, our results suggest that root allocation and directional placement might be uncoupled strategies that simultaneously provide stress tolerance and spatial responsiveness to neighbours, thus highlighting the importance of measuring both when studying belowground plant competition.

## 1. Introduction

Root placement in the soil has important implications for plant fitness in natural habitats because it can determine plants' ability to obtain nutrients and water, and also govern their competitive interactions with neighbouring plants [1–8]. Increasing evidence suggests that root placement and allocation are mediated by plants’ ability to perceive and integrate belowground cues regarding both nutrient availability and neighbour presence [9–14]. In particular, plants have been shown to respond to non-nutritional cues that provide complex information on their neighbours, including root exudates that indicate the genetic identity and relatedness of neighbours [15–21], as well as volatiles [22], and even aboveground cues that indicate the direction of belowground competition [23,24].

In response to the varying competition cues perceived belowground, plants were shown to demonstrate varying patterns of root placement and proliferation (reviewed in [1,25]). First, some studies have shown that under competition plants tend to exhibit a preemptive confrontational response, whereby they over-proliferate their roots and increase root biomass allocation [26–30], or aggregate their roots in the direction of neighbouring plants [9,14,31,32]. By contrast, other studies have shown that under competition plants tend to decrease biomass allocation to roots [17,33] or exhibit avoidance behaviour and grow their roots away from competing neighbours [12,13,34–36]. Finally, some plants were also found to exhibit no response to neighbours in root placement or proliferation [32,37–39], which could suggest that these species either lack the ability to detect neighbours, or that under some conditions, plants might employ alternative types of responses, such as increases in nutrient-uptake efficiency [40].

Inconsistencies in the responses of plants to belowground competition could be attributed to variations in the levels of genetic relatedness between plants and their neighbours, which could determine their cooperation levels [16,20,41]. Other studies have suggested that these inconsistencies could result from variations in the distribution of soil resources that could affect plants' ability to monopolize nutrient patches [9,14]; from variations in distances between neighbours and their resulting nutrient transportation costs within the plant [34]; or from variations in nutrient use and mobilization [14].

However, variations in belowground plant responses to competition might also stem from species-specific competition strategies [42], which might not always align with the way these responses are estimated. For example, some plants might respond to competition by investing in root proliferation (or biomass allocation to roots) [43], while others might change their root architecture and direct their roots towards or away from competing neighbours [1], without altering their overall biomass investment in root production. Thus, measuring both strategies might be crucial to understanding plant responses to belowground competition, but only seldom studies measured both simultaneously (but see [34,44]).

Different belowground responses to competition might also be attributed to variations in competitive hierarchies between plants and their neighbours. For example, previous studies on plant responses to aboveground competition have indicated that plants can assess the relative height of their neighbours, probably through sensing vertical gradients of increased light intensity and R:FR (the ratio of red to far-red light) next to neighbours [42,45–47]. Accordingly, plants have been shown to increase their vertical shade avoidance in response to short neighbours that can be easily outcompeted, but shift to shade tolerance responses under competition with tall neighbours [45]. Similarly, neighbour size belowground might be estimated in plants via different cues, such as the strength of nutrient-depletion gradients created around neighbours, as well as the concentration of root exudates they emit. As a result, plants might be able to assess the relative competitive ability of their neighbours and integrate this information in their belowground responses. For example, in a multi-species experiment, Lepik *et al*. [48] found a positive correlation between the extent of root aggregation between interspecific neighbours and their similarity in shoot biomass at harvest. However, to the best of our knowledge, no previous study has explicitly examined the effect of neighbour size on root-foraging decisions and belowground competitive strategies of plants.

In this study, we aimed to fill these knowledge gaps by examining the effects of neighbour size and density, as well as the distribution of soil resources on both root production and allocation and directional root placement. We used *Pisum sativum* as our focal plant and measured its responses to uniform versus patchy soil nutrient distribution, as well as to competition with *Festuca glauca*, which varied mainly in its root size (big versus small) and density (single versus double). We predicted that: (1) *P. sativum* plants will increase their directional root placement towards neighbours if these neighbours are small and can be easily outcompeted compared to bigger neighbours; (2) such root aggregation will be more pronounce under a patchy distribution of soil nutrients, which can be monopolized by plants; and (3) root biomass allocation of *P. sativum* will increase with neighbour size and density due to increases in the intensity of belowground competition they impose.

## 2. Material and methods

### (a) Focal and neighbour plants

As the focal plant, we chose the legume *Pisum sativum* (var. arvense Poir cultivar), which was previously shown to perceive and respond to belowground competition cues [26,35] (but see [39]). The cultivar grass *Festuca glauca* was chosen as the neighbour plant due to its dark roots, which can be easily distinguished from the white *P. sativum* roots. Moreover, *F. glauca* is a short tussock grass up to 18 cm tall that does not impose strong light competition on *P. sativum*, which grows tall up to 40 cm. However, due to its clumped clonal growth, *F. glauca* can be easily divided into clusters of varying diameters, thus generating neighbours that vary in their belowground competitive effect but not in their height and aboveground competition.

In order to create neighbour plants that differ in size, 20 *F. glauca* plants were purchased from a nursery, and in February 2022, non-rooting clumps of each plant were divided into groups of either 3–4 or 9–12 clones, representing small or big neighbours, respectively. The plants were then grown for two weeks in seeding trays filled with sand until they produced 5 cm roots.

### (b) Experimental setup

**Figure 1.**
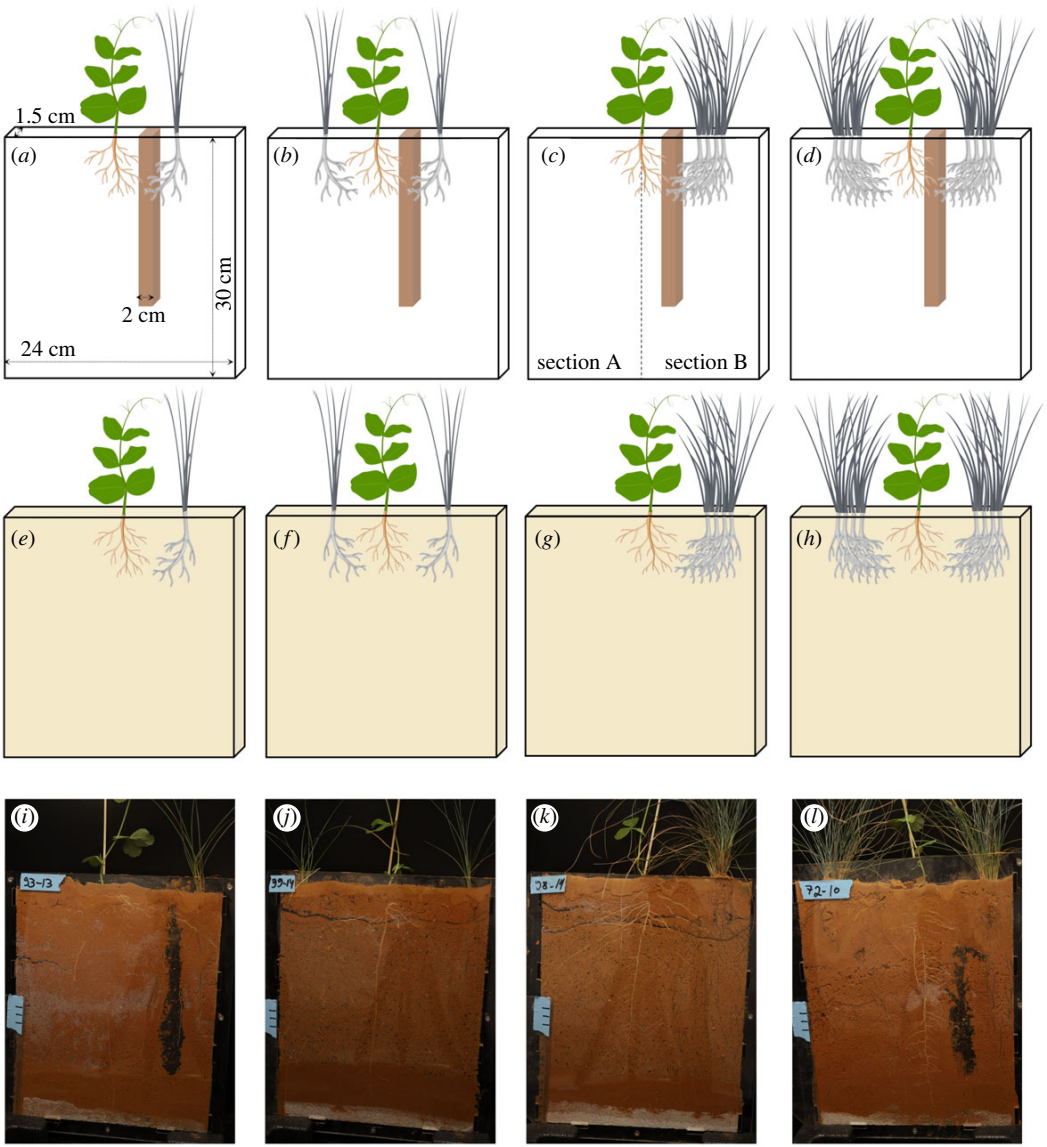
Schematic illustration of the experiment exploring the ability of *Pisum sativum* plants to integrate information on nutrient distribution, neighbour size and neighbour density in their spatial belowground responses. The target *P. sativum* plant (in green) was planted in the rhizobox centre. *Festuca glauca* was used as a neighbour plant (in grey) with either small (*a,b,e,f*) or big (*c,d,g,h*) sizes, and single (*a,c,e,g*) or double (*b,d,f,h*) densities. Soil nutrient distribution was either patchy (*a–d*, brown column) or uniform (*e–h*, yellow background). Single neighbours were located on the same rhizobox section as the nutrient patch (*a,c*). Included are pictures from the experiment of rhizoboxes in the patchy soil nutrient distribution with a single small (*i*) or double big (*l*) neighbours, and in the uniform soil nutrient distribution with double small (*j*) or a single big (*k*) neighbour.

In order to trace roots’ directional growth and to distinguish between focal and neighbour roots, the plants were grown in flat containers with a transparent front (‘rhizoboxes’; Vienna Scientific Instruments), which facilitate root observation by constraining their growth in two dimensions [30,49]. Each rhizobox was constructed of one black and one transparent Plexiglas plate (30 × 24 cm^2^) separated by thin spacers (1.5 cm) at the sides and bottom (figure 1*a*). A total of 160 rhizoboxes were filled with coarse sand and red loam at a 4 : 1 ratio as a background low-nutrient soil, and 50 ml of composting soil, which was either concentrated in a single column (20 × 2 × 1.5 cm^3^) (figure 1*a–d,i,l*) or uniformly mixed in the soil (figure 1*e–h,j,k*).

In February 2022, *P. sativum* seeds were germinated for 2 days in Petri dishes, following which a single *P. sativum* seedling was planted in the centre of each rhizobox and assigned to one of four combinations of neighbour size (a small or big *F. glauca* neighbour) and density (figure 1). In the low and high neighbourdensity treatments, *F. glauca* was planted on either one or both sides of *P. sativum*, respectively, halfway between *P. sativum* and the rhizobox edge (figure 1). In this setup, *P. sativum* plants under the patchy nutrient distribution were closer to the nutrient patch than one of their *F. glauca* neighbours in the double neighbour density. This setup allowed testing for the responses of *P. sativum* to neighbours of varying sizes, and according to their relative ability to monopolize a high-quality nutrient patch.

Each treatment was replicated 20 times, resulting in 160 rhizoboxes in total [2 nutrient distributions (uniform versus patchy) × 2 neighbour sizes (small versus big) × 2 neighbour densities (single versus double) × 20 replicates]. However, in 20 rhizoboxes, either the *P. sativum* seedling or *F. glauca* neighbour did not survive the transplant, leaving 140 rhizoboxes in total (16–19 replicates per treatment combination). All rhizoboxes were covered in opaque plastic sheets to prevent exposure to light and were tilted at a 30° angle to increase root growth along the transparent plate and enable its tracing. The plants were grown in a greenhouse at Tel Aviv University (full sunlight, 25°C) for 30 days, during which they were irrigated twice a week with 50 ml water. The pots were arranged on greenhouse benches in a randomized block design (eight treatment combinations per block).

### (c) Measured variables

At the end of the experiment, the spatial root distribution of *P. sativum* was estimated by photographing the transparent side of the rhizobox, using a Canon EOS-6Dii DSLR camera and a Canon 50 mm F2.5 lens in a photography tent. The images were used for analyses of total root length with the WinRhizo Tron software (Regents Instruments, Quebec, Canada), which allows for root measurements with a soil background. Directional root placement of *P. sativum* in the rhizobox was estimated per plant as the relative change in root length measured between two sections of the rhizobox: [(B − A) / (A + B) × 100], where A represents the rhizobox section without the nutrient patch and/or single neighbour, and B represents the rhizobox section with them (figure 1*c*). Negative and positive values indicate greater root distribution in section A and B, respectively, while zero values indicate even root distribution in both sections.

To examine our assumption of varying root competition imposed by neighbour size and density, total root length of *F. glauca* per rhizobox was analysed with the same images using the WinRhizo Tron software (see results in electronic supplementary material, figure S1 and electronic supplementary material, table S1).

Following photography of the rhizoboxes, the shoots of *P. Sativum* plants were cut, and their roots were finely washed. The biomass of the shoots and roots was weighed with an analytical balance (XSR105, Mettler-Toledo, Greifensee, Switzerland) after oven drying the samples at 60^o^ C for 3 days. Total root and shoot biomass were used to estimate root allocation of *P. sativum*, which was calculated as the ratio between root and shoot biomass.

### (d) Statistical analyses

The effects of nutrient distribution, neighbour size and density and their interactions on the different dependent variables were analysed using linear mixed models with block as a random factor. Shoot and root biomass and root length were log-transformed to meet model assumption of normal distribution. For analyses with significant effects, differences between treatment groups were analysed using a *post-hoc* pairwise least significant difference (LSD) test with the Benjamini–Hochberg false discovery rate correction [50]. These analyses were performed using IBM SPSS Statistics 29.

While adjustments in root allocation (root to shoot biomass ratio) can reflect optimal plastic responses to resource competition, they might also result from allometric constraints [51–54]. Hence, to separate between these two drivers of shifts in root allocation, standardized major axes (SMA) regression analyses were performed between root and shoot biomass, testing for equality in slopes and elevation (*y*-intercept) of this regression among different levels of nutrient distribution, neighbour size and neighbour density [55]. Root and shoot biomass were log-transformed prior to analysis. These analyses were performed using the SMATR package v. 3.4-8 [55] in R [56] and R-Studio v. 2023.9.1.494 [57].

## 3. Results

**Figure 2.**
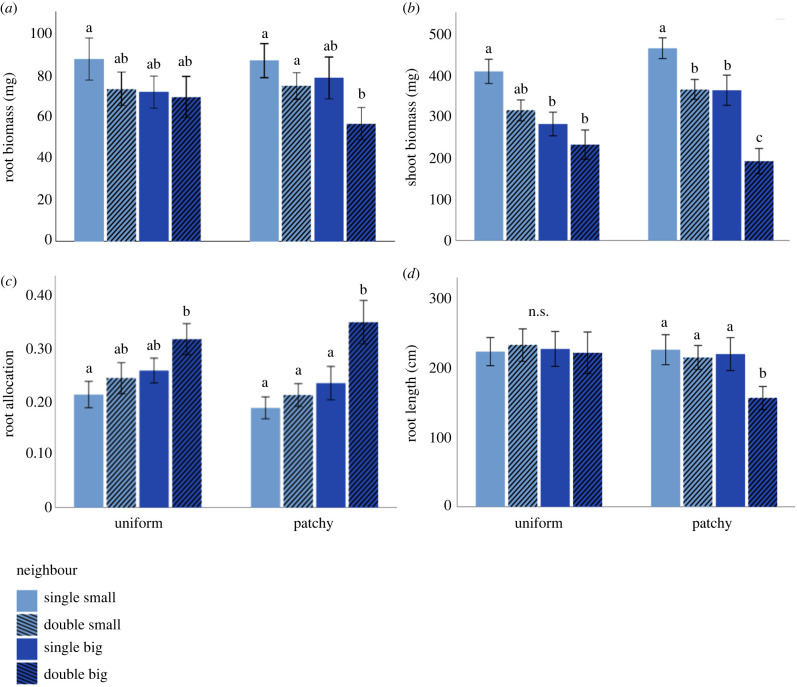
Responses (means ± s.e.) of *P. sativum* to nutrient distribution (uniform versus patchy) as well as the size (small versus big) and density (single versus double) of neighbouring *F. glauca*, in root biomass (*a*), shoot biomass (*b*), root allocation (root to shoot biomass) (*c*), and root length (*d*). Different lowercase letters indicate statistically significant pairwise comparisons within the uniform or patchy nutrient distribution treatments (with the Benjamini–Hochberg false discovery rate correction).

Biomass production of *Pisum sativum* was affected by both neighbour size and density but not by nutrient distribution in the soil. Specifically, root and shoot biomass were highest under competition with a single small *F. glauca* neighbour, lowest under competition with two big neighbours, and intermediate under competition with either a single-big or two-small *F. glauca* (table 1, figure 2*a,b*). Similarly, root biomass allocation (root to shoot ratio) was also affected by both neighbour size and density but not by soil nutrient distribution and was highest under competition with two big *F. glauca* neighbours and lowest under competition with a single small neighbour (table 1, figure 2*c*).

**Table 1.** Results of a linear mixed model for the effects of nutrient distribution, neighbour size and density, and their interactions on directional root placement, total root length, root allocation, root and shoot biomass, with block as a random factor. Significant effects are indicated in bold.

fixed effect	root biomass (mg)		shoot biomass (mg)		root allocation		total root length (cm)		directional root placement	
	*F*	*p*	*F*	*p*	*F*	*p*	*F*	*p*	*F*	*p*
nutrient distribution (*N*)	0.112	0.738	1.072	0.303	0.189	0.667	1.405	0.238	47.025	**<0.001**
neighbour size (*S*)	10.358	**0.002**	55.901	**<0.001**	18.166	**<0.001**	4.531	**0.035**	0.047	0.829
neighbour density (*D*)	8.819	**0.004**	43.920	**<0.001**	12.246	**<0.001**	2.867	0.093	5.351	**0.022**
*N* × *S*	0.388	0.535	1.761	0.187	2.636	0.107	1.451	0.231	0.248	0.619
*N* × *D*	0.436	0.510	3.893	0.051	2.080	0.152	1.247	0.261	5.315	**0.023**
*S* × *D*	0.645	0.424	6.055	**0.015**	3.841	0.052	4.137	**0.044**	0.434	0.511
*N* × *S* × *D*	3.932	0.050	4.192	**0.043**	0.928	0.337	1.175	0.281	0.080	0.778

**Figure 3.**
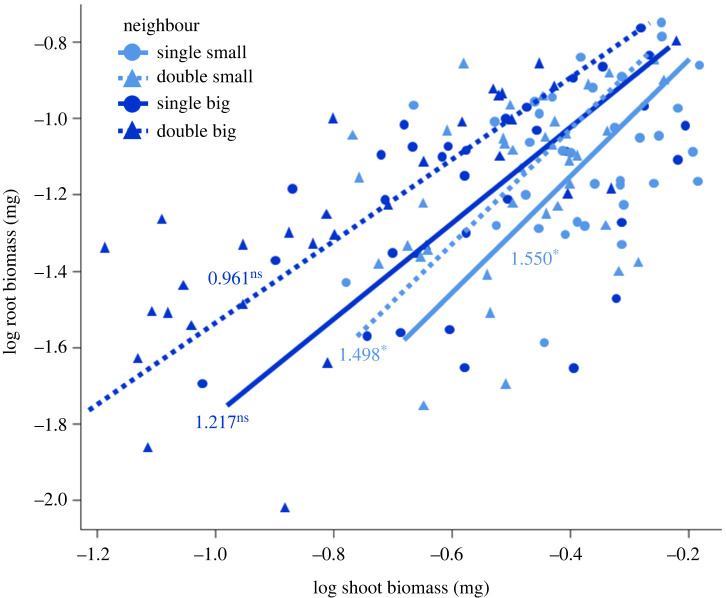
Standardized major axis (SMA) relationships between root and shoot biomass of *P. sativum*, according to the different treatment combinations of neighbour size and density (see table 2). Also shown are slope values for each treatment combination and their probability of equaling 1 (ns, *p* > 0.05; *, *p* < 0.05).

The positive effect of neighbour size and density on root allocation is also exhibited in the SMA results (table 2, figure 3). These results further suggest that the increase in root allocation exhibited by *P. sativum* in response to neighbour density is an adaptive plastic response rather than a result of allometric constraints, as indicated by the different elevations and similar slopes (table 2, figure 3). Similarly, the response to neighbour size represented adaptive plasticity, as shown by the different elevations (table 2, figure 3), although this response was also differently affected by plant size, as indicated by the different slopes (table 2, figure 3). However, the fact that in the two small-neighbour treatments, the slopes are higher than 1, suggests that in these treatments, bigger *P. sativum* plants allocated proportionally more biomass to roots (figure 3), and therefore these size-related effects are not due to allometric constraints (i.e. when smaller plants have lower root allocation), and might reflect decreased shoot allocation under competition with small neighbours.

**Table 2.** Standardized major axis (SMA) results for the effect of nutrient distribution, neighbour size and neighbour density on the relationships between root and shoot biomass, including tests for equality of slopes (LRT) and elevation (Wald statistics) (significant effects are shown in bold).

fixed effects	slope equality		elevation equality	
	LRT	*p*	Wald	*p*
nutrient distribution	1.244	0.265	0.615	0.433
neighbour size	4.132	**0.042**	13.320	**<0.001**
neighbour density	1.354	0.245	5.710	**0.017**

**Figure 4.**
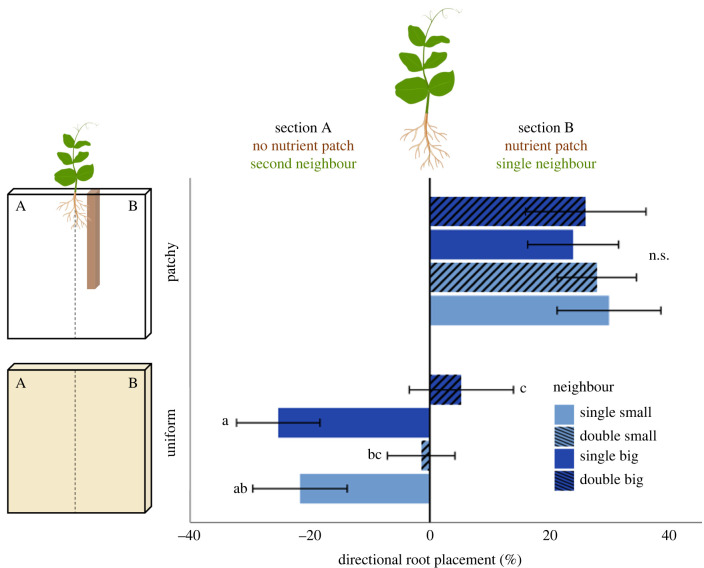
Responses (means ± SE) of *P. sativum* to nutrient distribution (uniform versus patchy), as well as the size (small versus big) and density (single versus double) of neighbouring *F. glauca*, in directional root placement in the rhizobox relative to its centre. The nutrient patch and single neighbour were located in section B of the rhizobox. Directional root placement was estimated per plant as the relative change in root length measured between sections A and B of the rhizobox: [(B − A) / (A + B) × 100]. Negative and positive values indicate greater root placement in section A and B, respectively, while zero values indicate even root placement in both sections. Different lowercase letters indicate statistically significant pair-wise comparisons within the uniform or patchy nutrient distribution treatments (with the Benjamini–Hochberg false discovery rate correction).

Total root length of *P. sativum* was affected by neighbour size and by its interaction with neighbour density and was lowest under competition with two big *F. glauca* neighbours (table 1, figure 2*d*). Unlike root biomass and total length, directional root placement of *P. sativum* in the rhizoboxes was affected by soil nutrient distribution (table 1, figure 4) and its interaction with neighbour density (table 1, figure 4). Specifically, when nutrients had a patchy distribution, the roots grew more toward the nutrient patch, regardless of neighbour density, but when nutrients were distributed uniformly, root growth was either uniform, when *F. glauca* were located on both sides, or away from *F. glauca* when it was single (table 1, figure 4). However, the directional root placement of *P. Sativum* was unaffected by neighbour size, with a single small *F. glauca* neighbour having the same effect as a big neighbour, and two small neighbours having the same effect as two big neighbours, under both nutrient distributions (table 1, figure 4).

## 4. Discussion

We studied the joint effect of neighbour size, neighbour density and the distribution of soil resources on root production and allocation and directional root placement in *P. sativum.* When *P. sativum* plants were subjected to big and double *F. glauca* neighbours, they exhibited decreased biomass production both above and belowground, but increased biomass allocation to the roots, and this response likely reflects adaptive plasticity rather than allometric constraints. By contrast, directional root placement was only affected by the spatial distribution of both *F. glauca* and the nutrients in the rhizoboxes. For example, when nutrients were patchily distributed, *P. sativum* always grew its roots in the direction of the nutrient patch, regardless of neighbour density or size, and when nutrients were distributed evenly, it grew its roots away from a single *F. glauca* neighbour.

Interestingly, our results indicate that the two responses—overall root biomass allocation and directional root placement—are uncoupled in *P. sativum*, which aggregated their roots towards neighbours while decreasing root biomass allocation, and vice versa—exhibited neighbour avoidance while increasing root allocation. These results therefore suggest that plants might be able to simultaneously employ two different competition strategies that are induced in response to different cues. First, increased root allocation, which enhances the uptake of limited resources [3,58–61], might be an indirect response to the overall resource limitation as a result of competition [43,62,63]. Root allocation can thus be considered a tolerance competition strategy, induced by low nutrient concentrations. By contrast, directional root placement may allow plants to respond spatially to the positioning of belowground resources and neighbours, and engage in either neighbour avoidance or aggregation [1,9,13,14,36], independently from the intensity of the competitive interaction they experience. This spatial response can be induced by cues that carry spatial information, such as gradients of increased nutrient concentrations away from neighbours, or in the direction of nutrient patches [2,3,36]. However, spatial cues that indicate neighbour presence can also be gradients of non-resource competition cues, such as root exudates, volatiles and aboveground changes in R : FR [15,16,20,22,23].

Similarly to the results of this study, Cabal *et al*. [34] found that plants tend to grow their roots away from competing neighbours, while simultaneously increasing local root proliferation. In this study, however, we show that *P. sativum* either avoided competition or grew its roots toward *F. glauca*, according to whether nutrients were distributed patchily or uniformly, respectively. These results are consistent with those of previous studies, which suggest that a competitive monopolization of belowground space is contingent on the availability of nutrient-rich soil patches [9,11–14]. Such an integrated effect of both neighbour presence and soil resource distribution can result from an additive response to their combined impact on nutrient availability. For example, depletion zones created around neighbouring roots are likely to have higher impact on nutrient availability when nutrients are uniform and low compared to when plants share access to nutrient-rich patches. However, in this study, bigger *F. glauca* neighbours, which probably created greater depletion zones, evident by their stronger negative effect on *P. sativum*, elicited the same root placement responses as small neighbours, regardless of whether nutrients were uniformly or patchily distributed. This result indicates that root distribution of *P. sativum* might not be mediated merely by neighbour effect on soil resource availability, but rather by an integrated response to additional non-resource competition cues, as suggested above.

In contrast to our predictions, we found that neighbour size had no effect on the directional root placement of *P. sativum*. Although to the best of our knowledge, our study is the first to examine the effect of neighbour size on belowground responses, previous studies have shown that neighbour size can affect aboveground plant responses to light competition; with shorter neighbours inducing increased preemptive vertical growth, and taller neighbours inducing greater shade tolerance [42,45–47]. This difference in responsiveness to neighbour size might be attributed to the fact that aboveground competition is often size-asymmetric due to the unidirectional nature of light, while belowground competition is mostly size-symmetric over soil resources that often lack directionality and are ephemeral [64,65]; but see [66]. Hence, neighbour size might not play an important role in determining the need to monopolize soil resources and thus might not affect directional root placement. Alternatively, the results of this study might suggest that despite the greater negative impact incurred to plants by bigger neighbours, they might not be able to assess the relative competitive ability and size of their neighbours belowground. For example, if neighbour presence is perceived by plants according to the occurrence and direction of root exudates but not by their concentrations, these exudates might not be able to provide information on the relative stature of neighbouring plants. Additional studies are therefore required to determine the mechanisms whereby non-resource cues are used by plants to estimate neighbour presence belowground.

This study has a few limitations that could hinder the generalization of its results. First, the responses exhibited by *P. sativum* might be specific to this target species as well as to *F. glauca* as a neighbour species. For example, Mobley *et al*. [39] combined the result of several intraspecific root competition experiments with *P. sativum* and found that root proliferation in this plant is likely unaffected by competition with conspecifics. Generalization of the responses found in this and other studies therefore requires the use of varying target species and varying competitor species (see for example [44]). Moreover, while both the target and neighbour species used in this study are cultivar model species, additional studies with wild target and neighbour plants that co-occur in nature might be more representative of the competitive interactions encountered by species in natural settings. Finally, due to the limited growth space in the rhizobox and the resulting short duration of the experiment, we were unable to measure the effect of the different competition treatments and the competitive strategies of *P. sativum* on its reproductive success. While this is a common limitation in these studies, it might hinder our ability to perceive additional allocation strategies used by plants in response to competition. For example, Chen *et al*. [33] found that *P. sativum* responded to belowground competition by decreasing root allocation, but increasing allocation to pods, suggesting that further studies should also examine the fitness and reproductive allocation outcomes of competition.

## 5. Conclusion

In this study we show that root allocation and directional root placement can be uncoupled strategies in plants, employed simultaneously to provide both stress tolerance and spatial responsiveness. Such a dual strategy can facilitate an optimal investment in competitive interactions, induced in response to both the intensity of these interactions and the spatial location of neighbouring plants. By studying both plastic responses, we were also able to show that while neighbour size affected root allocation, it had no effect on root avoidance or aggregation responses, which were more affected by the spatial distribution of nutrients in the soil. This result adds to a growing body of literature demonstrating that directional root placement is not mediated merely by neighbour effects on soil resource availability, but also by other non-resource cues that indicate neighbour presence. Future studies that will simultaneously investigate both competition strategies with additional species are needed to determine the generality of our results.

## Data Availability

Data available from the Dryad Digital Repository: https://doi.org/10.5061/dryad.cnp5hqcd5 [67]. The data are provided in electronic supplementary material [68].
